# Electromagnetic Wave-Absorption Properties of FDM-Printed Acrylonitrile–Styrene–Acrylate/Multi-Walled Carbon Nanotube Composite Structures

**DOI:** 10.3390/polym17152010

**Published:** 2025-07-23

**Authors:** Aobo Zhou, Yan Wang

**Affiliations:** School of Materials Science and Engineering, Wuhan Institute of Technology, Wuhan 430074, China; zab1218@163.com

**Keywords:** ASA, MWCNTs, fused deposition modeling, 3D printing, absorbing structure

## Abstract

The growing need for lightweight, customizable electromagnetic wave absorbers with weather resistance in aerospace and electromagnetic compatibility applications motivates this study, which addresses the limitations of conventional materials in simultaneously achieving structural efficiency, broadband absorption, and environmental durability. We propose a fused deposition modeling (FDM)-based approach for fabricating lightweight wave-absorbing structures using acrylonitrile-styrene-acrylate (ASA)/multi-walled carbon nanotube (MWCNT) composites. Results demonstrate that CST Studio Suite simulations reveal a minimum reflection loss of −18.16 dB and an effective absorption bandwidth (RL < −10 dB) of 3.75 GHz for the 2 mm-thick composite plate when the MWCNT content is 2%. Through FDM fabrication and structural optimization, significant performance enhancements are achieved: The gradient honeycomb design with larger dimensions achieved an effective absorption bandwidth of 6.56 GHz and a minimum reflection loss of −32.60 dB. Meanwhile, the stacked stake structure exhibited a broader effective absorption bandwidth of 10.58 GHz, with its lowest reflection loss reaching −22.82 dB. This research provides innovative approaches for developing and manufacturing tailored lightweight electromagnetic wave-absorbing structures, which could be valuable for aerospace stealth technology and electromagnetic compatibility solutions.

## 1. Introduction

The growing advancement of technology has led to escalating electromagnetic radiation (EMR) pollution caused by electronic equipment and smart infrastructure, which significantly endangers sustainable development, human health, and national data security [1,2,3,4]. Typically, electromagnetic wave-absorbing materials (EWAMs) attenuate and absorb incident electromagnetic waves (EMWs) by efficiently converting the energy of incident electromagnetic waves into heat or other dissipative forms. Notably, the ideal EWAMs are expected to possess critical features such as low weight, high absorption efficiency (high reflection loss), wide effective absorption bandwidth (EAB), and minimal thickness for ensuring excellent functionality in various application scenarios [5,6,7,8].

Conventional wave-absorbing materials, including ferrite and magnetic metal particles, have high magnetic loss capability, but their high density and susceptibility to oxidation limit their suitability for lightweight applications. On the other hand, dielectric materials such as carbon black and graphite, although having lower density, require higher filler content to achieve the desired absorption effect, which in turn increases the complexity and cost of fabrication [9,10]. Recently, multi-walled carbon nanotubes (MWCNTs) have gained significant attention as advanced electromagnetic wave-absorbing materials owing to their exceptional dielectric properties, high surface-to-volume ratio, and excellent charge transport characteristics. Typically, the 1D hollow tubular structure of multi-walled carbon nanotubes allows electrons to move freely along the axial direction, which endows them with excellent electrical and electromagnetic response properties. Furthermore, both theoretical and experimental studies have confirmed the excellent mechanical properties of carbon nanotubes, including high tensile modulus, strength, and elasticity. Even lower MWCNT contents (1.5–6.5% by weight) can significantly improve the dielectric constant and impedance matching of the composites [11,12]. For instance, Zhou et al. [13] found that incorporating 5 wt% MWCNTs into epoxy matrices dramatically boosts both electrical conductivity (by 10^9^ times) and R-band (26.5–40 GHz) electromagnetic absorption performance, which stems from the cooperative effects of interfacial polarization and charge relaxation mechanisms.

Nevertheless, traditional fabrication techniques for wave-absorbing structures face limitations in terms of shape complexity, dimensional tunability, and manufacturing accuracy. In contrast, 3D printing techniques, especially fused deposition modeling (FDM), can fabricate complex geometries with high accuracy and consistency, providing greater flexibility in the design and production of EWAMs [14,15,16,17,18,19]. Moreover, FDM also features a wide range of material compatibility, simplicity, cost-effectiveness, and production of mechanically robust components, giving it an advantage in the fabrication of customized absorptive structures [20,21,22]. For example, Lai et al. [23] fabricated single-layer conductive ABS wave-absorbing materials with multilayer microstructures using FDM. Their bionic “woodpile″ design (thickness: 3.5 mm) achieved an ultra-wide EAB of 5.43 GHz (RL < −10 dB) by optimizing dielectric impedance matching. Similarly, Zheng et al. [24] developed a gradient cellular EWAM using TPU/CIP composites via FDM, realizing a 3 mm-thick structure with an EAB of 4.64 GHz and a minimum reflection loss of −36.69 dB in the 8.48–13.12 GHz range. Subsequently, the pyramid architecture enhanced wave absorption across 15.88 GHz (2.12–18 GHz), reaching −49.75 dB minimum reflection loss and proving efficient electromagnetic coupling through structural design.

ASA is a high-performance thermoplastic resin consisting of an ASA graft copolymer blended with a styrene-acrylonitrile (SAN) random copolymer. While sharing similar mechanical properties with ABS resin, ASA exhibits superior weatherability and chemical stability due to its unique molecular structure [25,26]. Although PLA and ABS are the most commonly used thermoplastics in FDM due to their ease of processing and commercial availability, ASA has recently attracted increasing attention for functional composite applications. Yu et al. [27] found that incorporating CNTs in PLA composites via FDM had limited effects on mechanical properties, underscoring the importance of polymer-CNT compatibility for dispersion and performance. Both ABS and ASA contain polar acrylonitrile groups, which enhance interfacial bonding with CNTs and improve dispersion stability. The key structural difference lies in the elastomeric phase: ASA incorporates butyl acrylate elastomers instead of the butadiene elastomers found in ABS. This structural modification eliminates vulnerable carbon-carbon double bonds (C=C) in the polymer backbone, thereby significantly enhancing resistance to oxidative degradation [28,29,30]. Song et al. [31] demonstrated that ASA-based composites filled with graphite and carbon black exhibit excellent electrical conductivity, thermal stability, and electromagnetic interference-shielding effectiveness (EMI SE), thereby validating the theoretical feasibility of using ASA as a matrix for carbon-based functional absorbing materials. These exceptional characteristics, combined with outstanding dimensional stability and heat resistance (typically maintaining properties up to 85–100 °C), make ASA an ideal engineering plastic for outdoor applications where long-term environmental durability is required [32]. The material’s balanced properties portfolio has led to its widespread adoption in automotive components, building materials, and other applications demanding rigorous weathering performance.

Although many studies have investigated CNT-based microwave-absorbing materials, few have explored the use of ASA as a matrix for FDM 3D printing, especially in combination with MWCNTs to achieve tunable absorption and maintain good processability. Currently, research on ASA-based composites for the 3D printing of electromagnetic wave-absorbing structures remains limited. This study aims to address this gap by systematically investigating the design, fabrication, and performance of ASA/MWCNT composites specifically tailored for FDM-based microwave-absorption applications. Based on this research foundation, ASA was selected as the matrix material, with MWCNTs as the electromagnetic wave-attenuation medium to fabricate functional composites. Through FDM technology, these composites were processed into structured wave-absorbing components, exhibiting excellent microwave-absorption performance. This work establishes a systematic approach for designing and fabricating optimized EWAMs architectures via additive manufacturing, showing significant implementation prospects in engineering applications.

## 2. Materials and Methods

### 2.1. Materials

ASA (LI-911, LG Corporation, Seoul, Republic of Korea). MWCNTs (MCN4101, average pipe diameter 6–10 nm, average pipe length 30–60 μm, aspect ratio 3000–10,000, specific surface area 230–280 m^2^/g, Shenzhen Feimo Technology Co., Ltd., Shenzhen, China). Pentaerythritol tetra stearate (PETS, LG Corporation, Seoul, Republic of Korea).

### 2.2. Preparation of ASA/MWCNT Composite Materials

The ASA matrix was first dried at 80 °C for 6 h prior to processing. The dried ASA was then compounded with MWCNTs and a PETS flow modifier using a QE-70A mixer from Wuhan Qien Technology Development Co., Ltd., Wuhan, China. Melt blending was processed at 230 °C for 12 min with a rotational speed of 32 rpm. Composites A-2, A-4, A-6, and A-8 reflect the MWCNT percentages as outlined in Table 1.

### 2.3. Preparation of Coaxial Rings

**Figure 1:**
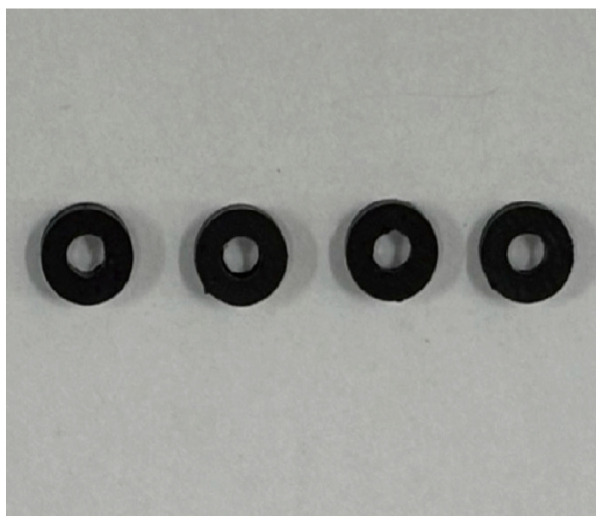
Schematic diagram of concentric annular specimens.

The electromagnetic characterization samples were prepared through compression molding using an R-3221 hot press (Wuhan Qion Technology Development Co., Ltd., Wuhan, China). The composite pellets were processed at 230 °C under 10 MPa pressure for 15 min, followed by controlled cooling to produce concentric annular specimens with dimensions of 3 mm inner diameter, 7 mm outer diameter, and 2 mm thickness, as shown in Figure 1.

### 2.4. Injection Molding of ASA/MWCNT Composites

Resistance measurement samples were prepared using a micro-injection molding machine (M-1200, Wuhan Qien Technology Development, Wuhan, China). Processing parameters were as follows: 235 °C melt temperature, 10 s mold closing, 16 s injection, and 4 s cooling. Straight specimens (80 × 10 × 4 mm^3^) were molded for electrical conductivity testing.

### 2.5. Preparation of ASA/MWCNT Filament Materials for 3D Printing

The composite filaments were prepared through a systematic process beginning with drying the ASA resin at 80 °C for 4–6 h. The dried resin was uniformly blended with MWCNTs before being processed through a TP-07 single-screw extruder (Dongguan Songhu Plastic Machinery Co., Ltd., Dongguan, China). Precise control of extrusion parameters—including a temperature profile ranging from 180 to 220 °C, screw rotation at 30–50 rpm, and take-up speed maintained at 0.8–1.2 m/min—yielded consistent filaments measuring 1.75 ± 0.05 mm in diameter.

### 2.6. FDM 3D Printing

**Figure 2:**
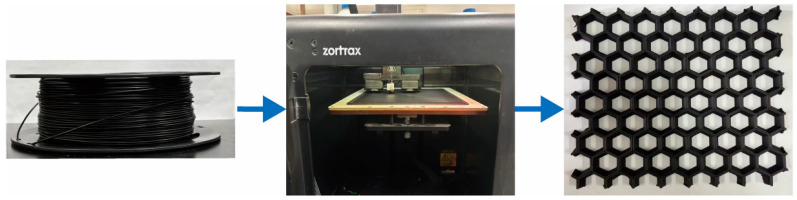
Schematic of the FDM process for manufacturing structures with tailored electromagnetic wave-absorption properties.

The FDM fabrication process was carried out using a Zortrax M200Plus 3D printer (Zortrax Company, Olsztyn, Poland) with the following optimized printing parameters: a nozzle temperature of 250 °C, build plate temperature of 100 °C, printing speed of 50 mm/s, and infill density of 30%. A schematic representation of the FDM process is provided in Figure 2.

### 2.7. Electromagnetic Simulation Software Calculation

The electromagnetic wave-absorption characteristics of the designed structure were systematically investigated using the commercial full-wave simulation software CST Studio Suite 2018. This advanced simulation platform provides comprehensive tools for analyzing complex electromagnetic problems. The simulation procedure was carefully implemented following several key steps to ensure accurate and reliable results. Firstly, the simulation environment was properly configured by establishing appropriate boundary conditions and material definitions. The boundary conditions were configured as follows: periodic conditions were applied along the x- and y-directions to simulate an infinite array, while open (radiation) boundaries were set in the z-direction for wave propagation. The Zmin boundary was defined as a perfect electric conductor (PEC) to model a reflective ground plane. Simulations employed the frequency-domain finite element method (FEM) with adaptive tetrahedral meshing, which was refined near material interfaces and high-field-gradient regions. A frequency-domain solver was selected as it is particularly suitable for analyzing steady-state electromagnetic wave propagation problems in the specified frequency range. The simulation covered the broad microwave frequency spectrum from 0 to 18 GHz, encompassing multiple important frequency bands, including S-band (2–4 GHz), C-band (4–8 GHz), X-band (8–12 GHz), and Ku-band (12–18 GHz). For the structural modeling, only a single unit cell of the periodic wave-absorbing structure was designed due to the periodic nature of the structure. The permittivity and permeability of ASA/MWCNTs were assigned based on the experimental data. Once the simulation is completed, the reflection loss characteristics can be analyzed from the 1D results.

### 2.8. Testing and Characterization

The ohmic resistance characteristics of the test samples A-2, A-4, A-6, and A-8 were measured using a high resistance meter (GT-1864, Dongguan High-speed Rail Detection Instrument Co., Ltd., Dongguan, China). The electrical conductivity (σ) was determined using the following relationship:

(1) $$\sigma =\frac{1}{\rho }=\frac{L}{RS}$$

Here, *R* represents the resistance obtained from measurements, *L* is the length of the straight bar linked to the high-resistance meter, *S* indicates its cross-sectional area, and Ω corresponds to the resistivity of the material.

The complex electromagnetic parameters of the A-2 to A-8 composites were characterized from 2–18 GHz using an N5224A vector network analyzer, while their melt flow properties were evaluated according to GB/T 3682-2018 standards (230 °C, 10 kg load). The electromagnetic wave-absorption performance was further assessed through reflection loss measurements employing the bow method across the same frequency range. The microstructure of ASA/MWCNT composites was examined using a field-emission scanning electron microscope (Sigma 360; ZEISS, Oberkochen, Germany) operating at 20 kV. Prior to imaging, the samples were cryogenically fractured in liquid nitrogen and sputter-coated with gold under vacuum.

## 3. Results and Discussion

### 3.1. Impact of MWCNT Loading on the Conductive Characteristics of ASA-Based Composites

From Table 2, the volume resistivity of ASA/MWCNT composites decreases significantly with the gradual increase of MWCNT content, while the electrical conductivity exhibits a dramatic jump from 10^−9^ to 10^−2^ S/cm. This behavior clearly demonstrates the formation of a continuous conductive network when MWCNT content exceeds a critical percolation threshold. The most significant conductivity transition occurs between 4% and 6% MWCNT loading, where conductivity increases by approximately four orders of magnitude (from 1.59 × 10^−4^ to 1.19 × 10^−2^ S/cm), indicating that the percolation threshold lies near 6% MWCNTs. Below this threshold (2–4%), the nanotubes remain largely isolated, resulting in limited electron transfer. Above 6%, although the conductive network becomes denser (as seen in the 8% sample), the rate of conductivity improvement decreases, suggesting network saturation effects. Such an abrupt change is primarily attributed to the overlapping effect between carbon nanotubes and improved dispersion efficiency, which establishes unobstructed electron transport pathways at the microscopic scale.

### 3.2. Influence of MWCNT Loading on Electromagnetic Parameter Characteristics of ASA Composites

Electromagnetic wave-attenuating ASA/MWCNT materials were prepared by the coaxial method, in which the structure-activity relationship between their dielectric properties and wave-absorbing performance was systematically analyzed. As no magnetic loss medium was added to the ASA/MWCNT composites, the real part (*μ*′) and imaginary part (*μ*″) of the relative permeability are 1 and 0, respectively, which do not vary with frequency.

**Figure 3:**
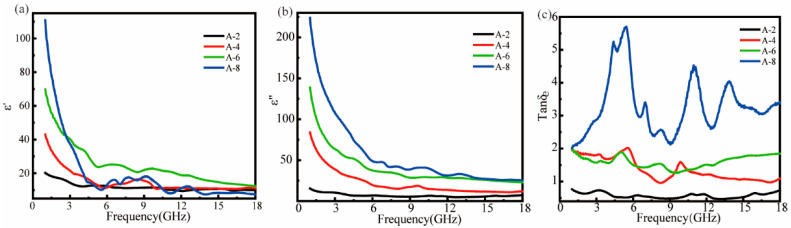
The *ε*′ (**a**), *ε*″ (**b**), and tan δe (**c**) of ASA/MWCNT composites.

Figure 3 presents the frequency-dependent dielectric properties (*ε*′ and *ε*″) of MWCNT-filled ASA composites across 1–18 GHz, obtained through coaxial-line measurements. The real (a) and imaginary (b) components of permittivity exhibit distinct variation trends with increasing MWCNT loading. Consequently, the dielectric constant of the composites increases significantly with the increase of MWCNT content, especially in the low-frequency region (<6 GHz), which exhibits strong interfacial polarization and conductive loss. Specifically, it stabilizes in the high-frequency region (greater than 10 GHz). It is noteworthy that *ε*′ tends to decrease with increasing frequency, while *ε*″ decreases first with frequency. Specifically, *ε*′ and *ε*″ represent the storage and dissipation capacity of electromagnetic energy, respectively [33]. When the frequency of the applied electromagnetic field is low, the electrons and dipoles have sufficient time to respond to changes in the electromagnetic field. However, the polarizability cannot be maintained as the external field frequency increases, leading to a decrease in the dielectric constant with increasing external field frequency [34].

The unexpected observation that the A-6 sample exhibits a higher *ε*′ than does A-8 (Figure 3a) can be explained by the electrical conductivity data in Table 2. Although A-8 contains more MWCNTs, its *ε*′ does not scale proportionally due to nanofiller agglomeration at higher concentrations, which reduces dispersion efficiency and active interfacial polarization sites. While A-6 shows a σ of 1.19 × 10^−2^ S/cm, A-8 reaches 3.68 × 10^−2^ S/cm, indicating better conductive network formation. However, this network saturation in A-8 suppresses polarization effects, limiting *ε*′ enhancement. The dielectric loss factor (tan δe, where δe = *ε*″/*ε*′) in Figure 3c reveals that A-8 exhibits three distinct loss peaks in the 3–6 GHz, 9–12 GHz, and 12–15 GHz ranges, demonstrating its multiband microwave-absorption capability. This tri-peak behavior suggests the coexistence of multiple polarization mechanisms, including interfacial polarization at lower frequencies and dipole relaxation processes at higher frequencies. However, the *ε*″ of samples A-6 and A-8 is significantly greater than *ε*′ due to excessive filling of the MWCNTs, which resulted in a mismatch between the *ε*′ and *ε*″, weakening the impedance-matching characteristics.

### 3.3. Simulation of Absorption Properties for ASA/MWCNT Composites

Multilayer interfacial reflection and transmission occur when a beam of electromagnetic waves of unit amplitude is incident vertically on a body of absorbing material, which can be understood by considering the electric and magnetic fields at each interface. Based on Kraus’ transmission line theory [35], the wave impedance (*Z_i_*) for each layer is determined by Equation (2), where *d_i_* denotes the *i*-th layer thickness, and *Z_i-_*_1_ represents the interfacial impedance between adjacent layers. The intrinsic impedance (*η_i_*) of the multilayer absorber is given by Equation (3), where *η*_0_ indicates the characteristic impedance in free space, and $\eta_{0}=\sqrt{\mu_{0}/\varepsilon_{0}}=1$. Moreover, *r_i_* denotes the intrinsic impedance parameter of multilayer absorbing material, which can be expressed as Equation (4). Among which, $\mu_{ri}$ and $\varepsilon_{ri\;}$ represent the $\mu_{r}$ and $\varepsilon_{r\;}$ (*ε*_r_ = *ε*′ *− jε″* and *μ*_r_ = *μ*′ − *jμ*″) of the *i*-th layer of multilayer material, respectively. Additionally, *c* stands for light velocity, and *f* indicates the frequency of EMWs, whereas *j* is an imaginary unit.

(2) $$Z_{i}=\eta_{i}\frac{Z_{i-1}+\eta_{i}\tanh\left(r_{i}d_{i}\right)}{\eta_{i}+Z_{i-1}\tanh\left(r_{i}d_{i}\right)}$$

(3) $$\eta_{i}=\eta_{0}\sqrt{\frac{\mu_{ri}}{\varepsilon_{ri}}}$$

(4) $$r_{i}=\frac{j2\pi f}{c}\sqrt{\mu_{ri}\varepsilon_{ri}}$$

The wave impedance calculation initiates with *Z*_0_ = 0 for the metal backing plate. For each subsequent layer (1 ≤ *I* ≤ n), *Zi* values are derived from Equation (2), ultimately enabling determination of the air-to-metamaterial interface reflection loss (RL). The electromagnetic wave RL of the absorbing material is expressed in dB, which indicates the loss capability of the material for a fixed frequency electromagnetic wave:

(5) $$RL=20\mathrm{lg}\left|\frac{Z_{n}-\eta_{0}}{Z_{n}+\eta_{0}}\right|$$

**Figure 4:**
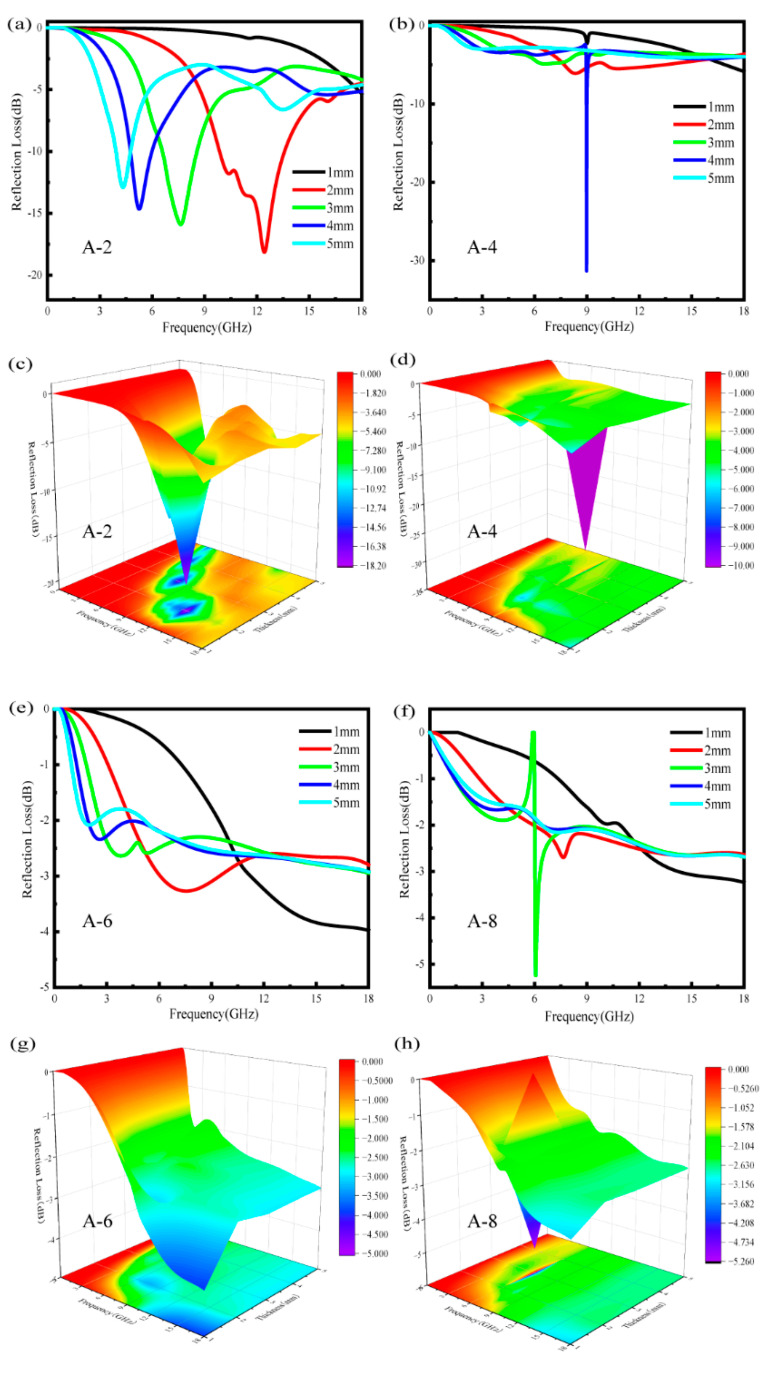
Theoretically calculated 3D and 2D RL spectra of ASA/MWCNT composites with different MWCNT content and thicknesses (1–5 mm). (**a**,**c**) Sample A-2. (**b**,**d**) Sample A-4. (**e**,**g**) Sample A-6. (**f**,**h**) Sample A-8.

Figure 4 shows the RL simulation results of ASA/MWCNT composites with different MWCNT contents (2–8%) at different thicknesses (1–5 mm). The composites containing 2% MWCNTs demonstrate superior electromagnetic wave-attenuation capabilities, achieving RL values exceeding −10 dB at specific frequency ranges, especially when the sample thickness reaches 2 mm or greater. For A-2, the excellent microwave-absorption performance is mainly attributed to the optimal combination of the dielectric loss mechanism with a favorable impedance-matching condition. The well-dispersed MWCNTs provide effective conductive loss and polarization relaxation, enhancing the dielectric loss. Meanwhile, the moderate permittivity of A-2 facilitates impedance matching with free space, as described by Equation (2). This ensures that more incident electromagnetic waves penetrate the absorber and are subsequently attenuated through dielectric loss, resulting in superior absorption performance. This enhanced absorption performance stems from an optimal combination of dielectric loss mechanisms and favorable impedance-matching conditions.

The RL peak exhibits a thickness-dependent low-frequency shift, following the quarter-wavelength (λ/4) interference cancellation principle [36]:

(6) $$d_{m}=\frac{nc}{4f_{m}\sqrt{\left|\left|\mu_{\gamma }\varepsilon_{\gamma }\right|\right|}}\left(n=1,3,5,\ldots \right)$$

where *d_m_* represents the material thickness and *f_m_* refers to the frequency of electromagnetic waves.

For A-4 at 9 GHz and A-8 at 6 GHz, the sharp absorption peaks primarily result from the λ/4 interference cancellation principle combined with impedance matching. When the material thickness approaches one-quarter of the electromagnetic wavelength at these specific frequencies, the phase difference between incident and reflected waves induces destructive interference, significantly improving absorption performance.

However, when the MWCNT concentration is elevated to 6–8%, the composites exhibit markedly different behavior. The substantial enhancement in electrical conductivity leads to deteriorated impedance-matching characteristics, consequently reducing the absorption efficiency. The effective absorption bandwidth becomes narrower, and the occurrence of significant RL minima becomes less frequent. This phenomenon occurs because highly conductive networks tend to reflect electromagnetic waves at the surface rather than facilitating their penetration and subsequent energy dissipation within the material. The three-dimensional RL representations vividly depict this transition, showing pronounced absorption peaks at lower MWCNT concentrations that gradually transform into attenuated, less distinct absorption profiles at higher filler contents. These findings underscore the importance of carefully optimizing both MWCNT concentration and composite thickness to attain maximum microwave-absorption efficiency in ASA/MWCNT systems.

Considering the electromagnetic wave-absorption performance of ASA/MWCNT composites, A-2 was chosen as the FDM material to print different structures for testing. Following comparative analysis of ASA/MWCNT systems, the A-2 variant demonstrated optimal characteristics for additive manufacturing of test structures to evaluate wave-absorbing behavior. Table 3 summarizes the theoretically calculated electromagnetic wave-absorption performance of the developed composites with different MWCNT contents.

### 3.4. Effect of MWCNT Content on MFR of ASA/MWCNT Composites

**Figure 5:**
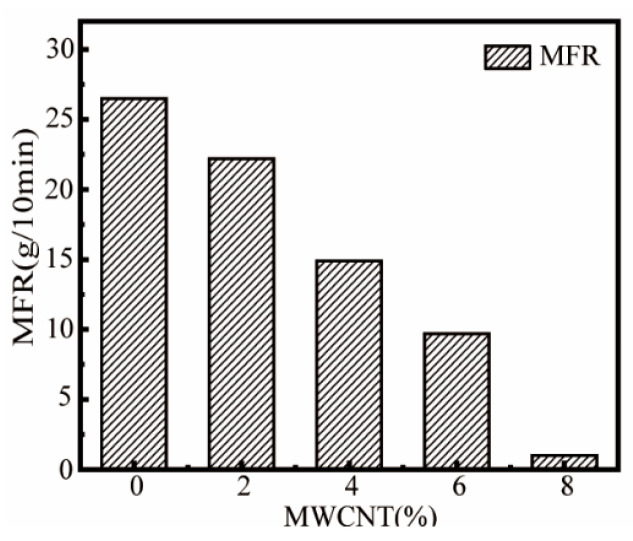
Effect of MWCNT content on MFR.

Figure 5 demonstrates that the MFR of ASA composites decreases significantly with the increasing content of MWCNTs, which is attributed to the rigid structure and high specific surface area of MWCNTs that limit the movement of polymer chains, resulting in an increase in melt viscosity. However, the MFR of the A-2 sample still reached 23 g/10 min, which was only 11% lower than that of pure ASA, indicating that it maintains excellent fluidity while realizing nanocomposite modification, which fully meets the processing requirements of FDM.

### 3.5. SEM Observation of MWCNT and ASA/MWCNT Composites

**Figure 6:**
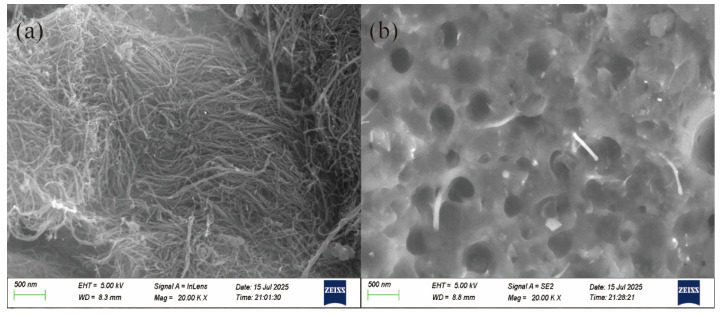
SEM images of (**a**) pristine MWCNTs and (**b**) A-2.

Figure 6 shows the morphology of MWCNTs and their dispersion in ASA-based composites. In Figure 6a, the SEM image of pristine MWCNTs reveals their characteristic slender, one-dimensional structure with smooth surfaces and high aspect ratios. However, their inherent entanglement often leads to agglomeration, making uniform dispersion within polymer matrices challenging. Figure 6b displays the cross-sectional morphology of the A-2 filament material for 3D printing, where the fractured surface reveals the dispersion state of the MWCNTs. A relatively uniform distribution is observed, with individual nanotubes embedded or protruding from the fracture surface. This improved dispersion is attributed to the strong shear forces generated by the single-screw extruder during processing, which effectively reduces agglomeration, promotes conductive network formation, and enhances the composite’s electromagnetic wave-absorption performance.

### 3.6. Electromagnetic Wave-Absorption Properties of 3D-Printed ASA/MWCNTs

**Figure 7:**
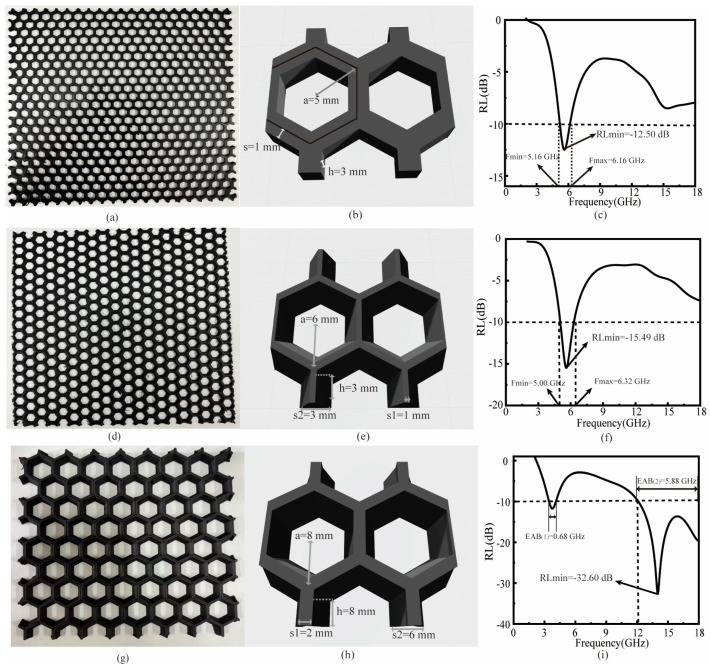
(**a**,**d**,**g**) 3D-printed sample of honeycomb structure, gradient-based honeycomb structure, and larger-sized gradient honeycomb structure. (**b**,**e**,**h**) Schematic diagram of unit structure. (**c**,**f**,**i**) Its RL at 2–18 GHz.

**Figure 8:**
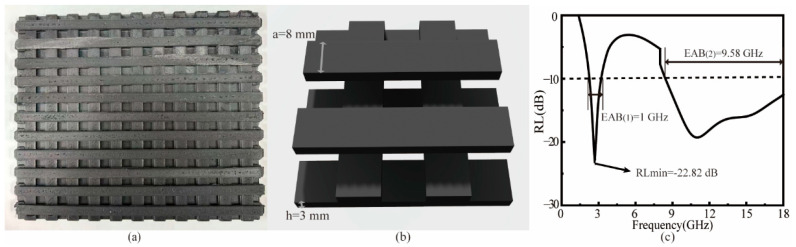
(**a**) 3D-printed sample of stacked wooden pile structure. (**b**) Schematic diagram of unit structure. (**c**) RL at 2–18 GHz.

ASA/MWCNT filaments were fabricated into uniform filaments with a diameter of 1.75 ± 0.05 mm by a single-screw extruder. As shown in Figure 7 and Figure 8, four metamaterial structures (180 mm × 180 mm) were printed by FDM, with each structure tested for wave absorption by the arch method.

#### 3.6.1. Honeycomb Structures

The honeycomb structure is a highly efficient absorber for electromagnetic waves, with excellent lightweight properties due to its porous topology. Periodically arranged hexagonal cells greatly increase the material’s specific surface area by more than a factor of three while minimizing weight. Such design enhances the interfacial interaction between the electromagnetic wave and the wave absorber, which induces multiple scattering from the pore walls, prolonging the wave propagation path and strengthening the loss mechanism. The combination of low density and optimized wave dissipation makes the honeycomb structure ideal for lightweight wave-absorbing materials.

A sample with honeycomb structure prepared with FDM technology is shown in Figure 7a–c. By controlling the fill rate to 30%, the structure is lightweight with a mass of only 35.17 g, which is 70% lighter than a solid plate structure of the same thickness. As a result, the structure achieves more than 90% energy dissipation of electromagnetic waves in a specific frequency band in the C-band (4–8 GHz), with an RLmin of −12.50 dB and an EAB covering the 1 GHz range. Remarkably, this high-porosity structure conforms to the performance requirements of modern electronic devices for wave-absorbing materials that are “thin, light, wide, and strong″.

A graded honeycomb architecture was developed through impedance-matching optimization (Figure 7d–f) to enhance conventional honeycomb absorbers’ performance. The electromagnetic loss characteristics of such structure in the 5.00–6.32 GHz range are significantly enhanced with an RLmin of −15.49 dB, which is 23.9% higher than that of the conventional honeycomb structure (−12.50 dB). By stratifying and adjusting the inclination angle of the unit structure, the gradient parameter design creates a progressive distribution of equivalent dielectric constants, which effectively improves the impedance-matching characteristics. Consequently, this structure achieves an EAB of 1.32 GHz in the C-band (5 GHz frequency domain), expanding the original honeycomb structure by 32 with a thickness of 3 mm.

For enhancing the wave-absorbing properties of the honeycomb structure, a gradient honeycomb structure was employed in this study, which was optimized by strategically enlarging the unit cell dimensions (Figure 7g–i). The optimized honeycomb structure has a side length of a = 8 mm, height of h = 8 mm, and gradient spacing of s1 = 2 mm/s2 = 6 mm. Typically, the optimized configuration demonstrates enhanced electromagnetic dissipation characteristics in the dual operating bands (3.44–4.12 GHz and 12.12–18.00 GHz) with an RLmin of −32.60 dB. Remarkably, the EAB extends to 6.56 GHz, covering almost the entire Ku-band.

When the unit cell size increases from 5 mm to 6 mm, RLmin decreases, indicating enhanced absorption performance. This improvement stems from the transition from a straight-walled honeycomb to a slanted-wall design with the same height, which optimizes the impedance gradient along the wave propagation direction and improves matching with free space. Further increasing the unit cell to 8 mm while maintaining the slanted design generates a new absorption band at 12–18 GHz due to the larger dimensions enabling higher-order resonances, extended multi-reflection paths enhancing dielectric loss, and increased thickness better satisfying the λ/4 condition for destructive interference. This results in a secondary RL min at 14.0 GHz (−32.60 dB). Notably, despite this additional high-frequency absorption, RLmin in the C-band slightly increases, reflecting degraded low-frequency absorption. This reduction is attributed to changes in effective permittivity and geometric parameters that marginally compromise C-band impedance matching.

Overall, these results have experimentally verified that the dimensional optimization of the gradient honeycomb structure can effectively improve wideband wave-absorption capability.

#### 3.6.2. Stacked Wooden Pile Structure

Furthermore, a three-layer wooden pile structure was designed with strip dimensions of a = 8 mm and h = 3 mm, as displayed in Figure 8b. The structure exhibits a minimum RL of −22.83 dB and an EAB of 10.58 GHz, covering the entire X- and Ku-band as well as most of the S-band (Figure 8c). The RL spectrum in Figure 8c exhibits dual-band absorption characteristics: a narrow-band peak at the S-band, originating from structural resonance effects where the periodic grooves induce λ/4 resonant cancellation through stacked wooden pile cavity modes, and a broadband absorption spanning the entire X- and Ku-bands, primarily attributed to the synergistic effects of MWCNT-enabled dielectric loss and multi-mode scattering from the grooved architecture. This dual-mechanism design achieves both frequency-selective and wideband microwave attenuation within a single absorber. This configuration demonstrates ideal characteristics for FDM-fabricated microwave absorbers, combining excellent printability with optimal electromagnetic wave-absorption performance.

Table 4 summarizes the electromagnetic wave-absorption performance of various structured composites developed in this study.

### 3.7. Analysis of EWA Principles

**Figure 9:**
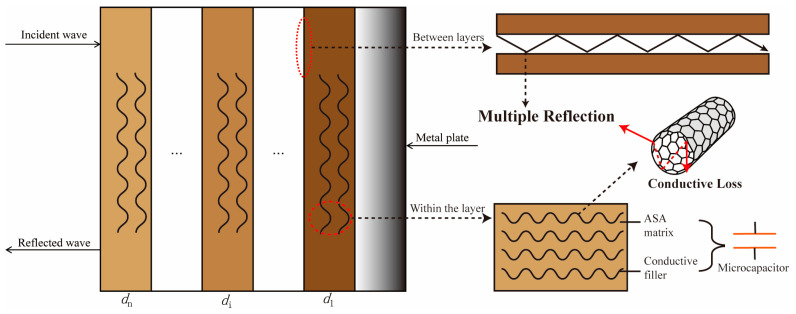
Schematic diagram of multi-layer absorbing wave structure.

Based on FDM 3D-printing technology and layer-by-layer stacking, the sample can be regarded as a multilayer absorbing structure, as shown in Figure 9. After the incident electromagnetic wave is partially reflected on the surface, the remaining energy penetrates into the layers. Due to the different dielectric parameters between the material and the air, the interlayer interface leads to an impedance mismatch, which triggers multiple reflections to extend the propagation path of the electromagnetic wave, thereby enhancing the energy loss frequency. The high intrinsic conductivity of MWCNTs enables them to form localized conductive regions in the ASA matrix to dissipate electromagnetic energy through interfacial polarization and dipole relaxation (induced by nanotube defects or functional groups). In addition, the insulating ASA matrix and conductive fillers create a micro capacitive effect. The nanoscale gaps between the MWCNTs form a distributed micro-capacitive network that consumes the energy of high-frequency electric fields by a charge accumulation-release process. Moreover, the electromagnetic energy can also be dissipated through micro capacitor-induced charge accumulation/release cycles, where hysteresis effects convert incident wave energy into thermal losses [37]. The synergistic interaction of these multi-scale dissipation phenomena, enabled by the meticulously engineered stratified morphology, yields effective wideband electromagnetic wave-attenuation characteristics.

## 4. Conclusions

The ASA/MWCNT composites exhibit the best electromagnetic wave-absorption when the MWCNT content is 2% while maintaining a high MFR of 23 g/10 min, completely satisfying the processing requirements of FDM. At a thickness of 3 mm, the printed common honeycomb structure achieves an absorption performance of RLmin −12.50 dB and an EAB of 1 GHz. In accordance with the gradient impedance-matching principle, the gradient honeycomb structure was designed to further improve the wave-absorbing performance (RLmin = −15.49 dB, EAB = 1.32 GHz). Both honeycomb structures achieve more than 90% absorption of electromagnetic waves in the C-band portion of the frequency domain and are also lightweight. By optimizing the unit size of the gradient cellular structure, the broadband absorption of the larger-size gradient cellular structure is significantly enhanced, with RLmin (−32.60 dB) and EAB (6.56 GHz) covering 36.3% of 0–18 GHz. In addition, the EAB of the stacked woodpile structure is 10.58 GHz, which covers the entire X- and Ku-bands, as well as most of the S-band, with a minimum RL of −22.83 dB, showing its optimal suitability as an FDM fabricated microwave-absorbing structure. Overall, the excellent weatherability of the ASA matrix for complex environments provides innovative insights into the design of novel lightweight absorptive materials.

## Figures and Tables

**Table 1:** Description of ASA/MWCNTs.

Sample	ASA (wt.%)	MWCNTs (wt.%)	PETS (wt.%)
A-2	96	2	2
A-4	94	4	2
A-6	92	6	2
A-8	90	8	2

**Table 2:** Electroconductive characteristics of MWCNT-reinforced ASA composites.

Sample	MWCNTs (wt.%)	ρ (Ω·cm)	σ (S/cm)
A-2	2	3.08 × 10^8^	3.25 × 10^−9^
A-4	4	6.29 × 10^3^	1.59 × 10^−4^
A-6	6	8.40 × 10^1^	1.19 × 10^−2^
A-8	8	2.70 × 10^1^	3.68 × 10^−2^

**Table 3:** Electromagnetic wave-absorption performance.

Sample	RLmin (dB)	EABmax (GHz) (RL < −10 dB)
A-2	−18.15	3.74
A-4	−31.31	0.04
A-6	−3.98	0
A-8	−5.22	0

**Table 4:** Different structures and their electromagnetic wave-absorption performance.

Sample	RLmin (dB)	EABmax (GHz) (RL < −10 dB)
Honeycomb structure	−12.50	1.00
Gradient-based honeycomb structure	−15.49	1.32
Larger-sized gradient honeycomb structure	−32.60	6.56
Stacked wooden pile structure	−22.82	10.58

## Data Availability

The dataset will be made available on request from the authors.

## Abbreviations

The following abbreviations are used in this manuscript:

FDM	Fused deposition modeling
ASA	Acrylonitrile-styrene-acrylate
MWCNT	Multi-walled carbon nanotube
EMR	Escalating electromagnetic radiation
EMWs	Electromagnetic waves
EWAMs	Electromagnetic wave-absorbing materials
EAB	Wide effective absorption bandwidth
RL	Reflection loss

## References

[1] Sun H., Che R.C., You X., Jiang Y.S., Yang Z.B., Deng J., Qiu L.B., Peng H.S. (2014). Cross-Stacking Aligned Carbon-Nanotube Films to Tune Microwave Absorption Frequencies and Increase Absorption Intensities. Adv. Mater..

[2] Tian W., Zhang X.Z., Guo Y., Mu C.H., Zhou P.H., Yin L.J., Zhang L.B., Zhang L., Lu H.P., Jian X. (2021). Hybrid silica-carbon bilayers anchoring on FeSiAl surface with bifunctions of enhanced anti-corrosion and microwave absorption. Carbon.

[3] Fang Y.S., Yuan J., Liu T.T., Wang Q.Q., Cao W.Q., Cao M.S. (2023). Clipping electron transport and polarization relaxation of Ti_3_C_2_T_x_ based nanocomposites towards multifunction. Carbon.

[4] Wu L.P., Gao H., Guo R.H., Li W.J., Wu F., Tao S.F., Zhu X.F., Xie A.M. (2023). MnO_2_ Intercalation-Guided impedance tuning of Carbon/Polypyrrole double conductive layers for electromagnetic wave absorption. Chem. Eng. J..

[5] Deng S., Jiang J., Wu D., He Q., Wang Y. (2023). Three-dimensional conductive network constructed by in-situ preparation of sea urchin-like NiFe_2_O_4_ in expanded graphite for efficient microwave absorption. J. Colloid Interface Sci..

[6] Hu X., Quan B., Ai B., Sheng M., Liu S., Huang X., Wu H., Lu X., Qu J. (2023). Engineering asymmetric multifunctional phase change composites for improved electromagnetic interference shielding and wireless personal thermal therapy. J. Mater. Chem. A.

[7] Tian Y., Zhi D., Li T., Li J., Li J., Xu Z., Kang W., Meng F. (2023). Graphene-based aerogel microspheres with annual ring-like structures for broadband electromagnetic attenuation. Chem. Eng. J..

[8] Yang L., Wang Y., Lu Z., Cheng R., Wang N., Li Y. (2023). Construction of multi-dimensional NiCo/C/CNT/rGO aerogel by MOF derivative for efficient microwave absorption. Carbon.

[9] Qin F., Brosseau C. (2012). A review and analysis of microwave absorption in polymer composites filled with carbonaceous particles. J. Appl. Phys..

[10] Meng F., Wang H., Huang F., Guo Y., Wang Z., Hui D., Zhou Z. (2018). Graphene-based microwave absorbing composites: A review and prospective. Compos. Part B-Eng..

[11] Pullen A., Zhao G.L., Bagayoko D., Yang L. (2005). Structural, elastic, and electronic properties of deformed carbon nanotubes under uniaxial strain. Phys. Rev. B.

[12] Coleman J.N., Khan U., Blau W.J., Gun’ko Y.K. (2006). Small but strong: A review of the mechanical properties of carbon nanotube–polymer composites. Carbon.

[13] Wang Z., Zhao G.-L. (2014). Electromagnetic wave absorption of multi-walled carbon nanotube-epoxy composites in the R band. J. Mater. Chem. C.

[14] Buswell R.A., de Silva W.R.L., Jones S.Z., Dirrenberger J. (2018). 3D printing using concrete extrusion: A roadmap for research. Cem. Concr. Res..

[15] Yuan Q., Li Z., Zhou D., Huang T., Huang H., Jiao D., Shi C. (2019). A feasible method for measuring the buildability of fresh 3D printing mortar. Constr. Build. Mater..

[16] Ma M., Gu J., Wang D.-A., Bi S., Liu R., Zhang X., Yang J., Zhang Y. (2023). Applications of 3D printing in aging. Int. J. Bioprinting.

[17] Sun Z., Zeng X., Deng X., Zhang X., Zhang Y. (2023). Droplet interface in additive manufacturing: From process to application. Droplet.

[18] Chen Y., Bi S., Gu J., Che Q., Liu R., Li W., Dai T., Wang D., Zhang X., Zhang Y. (2024). Achieving personalized nutrition for patients with diabetic complications via 3D food printing. Int. J. Bioprinting.

[19] Fu X., Gu J., Ma M., Liu R., Bi S., Zhang X., Zhang Y. (2024). Unique benefits and challenges of 3D-printed microneedles. Int. J. Bioprinting.

[20] Mohamed O.A., Masood S.H., Bhowmik J.L. (2015). Optimization of fused deposition modeling process parameters: A review of current research and future prospects. Adv. Manuf..

[21] Jiang W., Yan L., Ma H., Fan Y., Wang J., Feng M., Qu S. (2018). Electromagnetic wave absorption and compressive behavior of a three-dimensional metamaterial absorber based on 3D printed honeycomb. Sci. Rep..

[22] Rajan K., Samykano M., Kadirgama K., Harun W.S.W., Rahman M.M. (2022). Fused deposition modeling: Process, materials, parameters, properties, and applications. Int. J. Adv. Manuf. Technol..

[23] Lai W.W., Wang Y., He J.K. (2020). Electromagnetic Wave Absorption Properties of Structural Conductive ABS Fabricated by Fused Deposition Modeling. Polymers.

[24] Zheng Y., Wang Y. (2022). Electromagnetic-Wave Absorption Properties of 3D-Printed Thermoplastic Polyurethane/Carbonyl Iron Powder Composites. Polymers.

[25] Zhang Y., Xu Y., Song Y., Zheng Q. (2013). Study of poly(vinyl chloride)/acrylonitrile–styrene–acrylate blends for compatibility, toughness, thermal stability and UV irradiation resistance. J. Appl. Polym. Sci..

[26] Zhang X., Zhang J. (2018). Effect of core–shell structures of acrylonitrile–styrene–acrylate (ASA) terpolymer on the properties of poly(vinyl chloride) (PVC)/ASA blends: Miscibility, toughness, and heat resistance. J. Appl. Polym. Sci..

[27] Yu W.W., Zhang J., Wu J.R., Wang X.Z., Deng Y.H. (2017). Incorporation of graphitic nano-filler and poly(lactic acid) in fused deposition modeling. J. Appl. Polym. Sci..

[28] Rimdusit S., Damrongsakkul S., Wongmanit P., Saramas D., Tiptipakorn S. (2011). Characterization of coconut fiber-filled polyvinyl chloride/acrylonitrile styrene acrylate blends. J. Reinf. Plast. Compos..

[29] Blazey S.D. (2015). Formulation strategy to achieve highly colorable and weatherable ASA. Plast. Eng..

[30] Qi R., He C., Jin Q.J.B. (2019). Effect of acrylate-styrene-acrylonitrile on the aging properties of eucalyptus/PVC wood-plastic composites. BioResources.

[31] Song J.B., Yang W.B., Liu X.S., Zhang W.B., Zhang Y.H. (2016). ASA/graphite/carbon black composites with improved EMI SE, conductivity and heat resistance properties. Iran. Polym. J..

[32] Camposeco-Negrete C. (2020). Optimization of printing parameters in fused deposition modeling for improving part quality and process sustainability. Int. J. Adv. Manuf. Technol..

[33] Yang Z., Liang Q., Duan Y., Li Z., Li D., Cao Y. (2021). A 3D-printed lightweight broadband electromagnetic absorbing metastructure with preserved high-temperature mechanical property. Compos. Struct..

[34] Sobha A.P., Sreekala P.S., Narayanankutty S.K. (2017). Electrical, thermal, mechanical and electromagnetic interference shielding properties of PANI/FMWCNT/TPU composites. Prog. Org. Coat..

[35] Kraus J.D. (1948). Helical Beam Antennas for Wide-Band Applications. Proc. IRE.

[36] Li W., Wu T., Wang W., Zhai P., Guan J. (2014). Broadband patterned magnetic microwave absorber. J. Appl. Phys..

[37] Wang C., Li J., Guo S. (2019). High-performance electromagnetic wave absorption by designing the multilayer graphene/thermoplastic polyurethane porous composites with gradient foam ratio structure. Compos. Part A-Appl. Sci. Manuf..

